# Organic Amendments promote saline-alkali soil desalinization and enhance maize growth

**DOI:** 10.3389/fpls.2023.1177209

**Published:** 2023-08-24

**Authors:** Yaqi Wang, Ming Gao, Heting Chen, Yiwen Chen, Lei Wang, Rui Wang

**Affiliations:** ^1^ School of Agriculture, Ningxia University, Yinchuan, Ningxia, China; ^2^ School of Ecology and Environment, Ningxia University, Yinchuan, Ningxia, China

**Keywords:** organic amendment, saline–alkali soil, maize growth, maize nutrient utilization, and maize yield

## Abstract

Secondary soil salinization in arid and semi-arid regions is a serious problem that severely hampers local agricultural productivity and poses a threat to the long-term sustainability of food production. the utilization of organic soil amendments presents a promising approach to mitigate yield losses and promote sustainable agricultural production in saline-alkali soil. In this study, we established four distinct treatments, chemical fertilizer (CK), humic acid with chemical fertilizer (HA), carboxymethyl cellulose with chemical fertilizer (CMC), and amino acid with chemical fertilizer (AA), to elucidate their respective impacts on the reclamation of saline soil and the growth of maize. The findings of our study reveal notable variations in desalination rates within the 0-40 cm soil layer due to the application of distinct soil amendments, ranging from 11.66% to 37.17%. Moreover, application of amendments significantly increased the percentage of soil macro-aggregates as compared to the CK treatment. Furthermore, HA and AA treatments significantly augmented soil nutrient content (HA: 48.07%; AA: 39.50%), net photosynthetic rate (HA: 12.68%; AA: 13.94%), intercellular CO_2_ concentration (HA: 57.20%; AA: 35.93%) and maize yield (HA:18.32%; AA:16.81%). Correlation analysis and structural equation modeling unveiled diverse mechanisms of yield enhancement for HA, CMC, and AA treatments. HA enhanced yield by increasing organic matter and promoting soil aggregate formation, CMC improved soil water content and facilitated salt leaching due to its excellent water-holding properties, while AA increased yield by elevating soil organic matter and effective nitrogen content. Among the array of soil amendment materials scrutinized, HA treatment emerged as the most promising agent for enhancing soil conditions and is thus recommended as the preferred choice for treating local saline soils.

## 1 Introduction

In recent years, secondary soil salinization has emerged as a particularly serious global environmental problem that can be attributed to both natural factors and human activities, and is considered one of the main environmental risks (Pitman and Läuchli, 2002). At present, it is estimated that globally, approximately 1 billion hectares of land is salinized to various degrees, accounting for approximately 10% of the world’s total arable land. Moreover, the area of land affected continues to grow annually at a rate of 1.5–2.5 × 10^5^ ha (Mustafa et al., 2019). By 2050, it is predicted that approximately 50% of the world’s arable land will be adversely affected by salinization (Kumar and Sharma, 2020). A region that has succumbed to the detrimental effects of salinization is northern Ningxia in Northwest China. With its flat terrain, good soil quality, and the benefit of irrigation from the Yellow River, this is an important agricultural area and commercial grain production base in China. However, as a consequence of low precipitation and excessive evaporation, long-term continuous irrigation by the Yellow River has raised the groundwater levels, resulting in the accumulation of salt in the groundwater, along with surface transpiration, thereby contributing to serious secondary salinization of soil (Xiong et al., 1996). Given the typical characteristics of salinized soil, such as high salt concentration and strong alkali properties, hardening, and low productivity, large areas of saline–alkali land have been abandoned, leading to a further contraction of cultivated land resources and reductions in grain harvest, which are substantially restricting local agricultural development (Munns and Gilliham, 2015). Consequently, the management of saline–alkali land is of paramount importance in order to mitigate yield losses due to environmental stress and to achieve sustainable production with limited production inputs. To this end, we must take measures to promote the rational development and improved utilization of land, and optimize land management with a view to improving the comprehensive productivity of agriculture in saline–alkali lands, increasing the effective cultivated land area, and ensuring food production and food security.

In order to reduce and eliminate the adverse effects of soil salinization on crop growth and soil properties, various countermeasures have been implemented to improve soil quality and enhance land productivity. Some studies (D'hose et al., 2020; Wang et al., 2021), for example, have shown that the application of organic soil amendments can be an effective management approach for improving the productivity of farmland, particularly in regions characterized by poor-quality soils, such as saline–alkali soils. Among such materials applied as soil amendments, Humic acid (HA) is an organic colloid with strong cation adsorption and exchange capacities, which can effectively improve soil structure, promote the formation of soil agglomerate structure, improve soil organic matter, reduce soil salinity and alkalinity, and promote crop growth (Yang and Antonietti, 2020; De Castro et al., 2021). It is accordingly considered an excellent soil conditioner. Other materials include carboxymethyl cellulose (CMC), a cellulose ether derivative with a carboxymethyl structure, which can be obtained from natural cellulose modified via alkylation and etherification (Rahman et al., 2021). It has the advantages of being odorless, tasteless, non-toxic, readily soluble in water, and characterized by good photothermal stability (Zhang et al., 2022). In particular, its aqueous solution is noted for is diverse range of beneficial attributes, including its thickening, film formation, suspension, adhesion, and water-holding properties, and is accordingly particularly suitable for use as a water-retaining modifier in saline–alkali soils (Fu et al., 2023). In addition, amino acid (AA) fermentation tail liquor, produced during the production of monosodium glutamate, is a rich source of inorganic nutrients, amino acids, sugars, and other organic components, and can thus serve as an inexpensive readily available high-quality raw fertilizer material. Indeed, it is gradually emerging as a novel type of soil conditioner, on account of its ability to effectively improve soil physical and chemical properties and microbiological conditions. However, most previous studies (Wang et al., 2014; Page-Dumroese et al., 2018) on these amendments have focused on the physical and chemical properties of soils, and there is a paucity of research on their effects on water and salt transport in saline soils, especially on the adaptive characteristics and response of maize to soil amendment application on saline soils. Consequently, there is an immediate necessity to examine the impact of various soil amendment techniques on the process of desalination and maize growth in saline–alkali lands.

In this study, a field experiment was conducted on a salinized soil located in the northern region of Ningxia. The primary objective was to scrutinize the impact engendered by the application of HA, CMC, and AA on soil desalination and the growth of maize (*Zea mays* L.). To ascertain the efficacy of these amendments in ameliorating soil water content and abating salinity levels, several crucial metrics were carefully selected for evaluation. These included soil moisture, Electrical Conductivity (EC_e_), pH, and Sodium Adsorption Ratio (SAR_e_), as they hold paramount significance in influencing crop growth, especially in saline-alkali conditions. Furthermore, a comprehensive assessment of the soil aggregate structure and the abundance of available nutrients, such as alkali-hydrolyzable nitrogen (AN), available phosphorus (AP), available potassium (AK), and organic carbon (OC), was performed. The presence and availability of these vital nutrients influence soil fertility and directly impact the nutrient uptake capabilities of plants. An analysis of photosynthetic indices, water use efficiency, maize yield, and quality indices was undertaken. These multifaceted parameters provided an all-encompassing evaluation of the diverse effects induced by the distinct soil amendments on crop growth and productivity. To unravel the intricate interrelationships existing among the analyzed indicators under varying amendment conditions, both correlation analysis and structural equation modeling were adroitly employed. The outcomes of this research endeavor are poised to furnish a theoretical underpinning and technical guidance for the sustainable development and judicious exploitation of saline-alkaline land within the esteemed Yellow River Irrigation Area.

## 2 Materials and methods

### 2.1 Site description

This study was carried out at the Saline Land Water-saving and Salt-control Technology Demonstration Area (36°15’N, 106°25’E, 1100 m) in Pingluo County, Ningxia Hui Autonomous Region, Northwest China. The designated research site is ensconced in a locale distinguished by a temperate continental semi-arid climate, boasting an annual mean temperature of 9.4°C, encompassing an amplitude encompassing temperature extrema spanning from -22°C to 38.8°C. The effective accumulated temperature is between 3100 and 3300°C and the difference between day and night temperatures ranges between 10 and 15°C. Furthermore, the research site experiences a copious supply of solar radiance, with annual sunshine hours fluctuating between 2800 and 3000 hours, representing approximately 68% of daylight exposure. In concurrence, the study area encounters an average annual precipitation regime varying from 190 to 210 mm, while succumbing to an annual evaporation quotient of 1900 to 2100 mm, generating aridity in the environment. Nonetheless, the interplay of precipitation and evapotranspiration blesses the region with an average of 160 to 170 frost-free days per annum, essential for the successful growth and maturation of maize crops. Notably, the soil medium hosting the maize plantation is characterized by a saline-irrigated silt with a medium loam texture, ascertained to harbor integral physical and chemical properties pivotal to crop development, meticulously outlined in **Table 1**.

**Table 1:** The initial basic physical and chemical properties of the study site soil.

Soil layer	Bulk density	pH	EC_e_	SAR_e_
(cm)	(g cm^-3^)		(dS m^-1^)	(mmol L^-1^)
0-10	1.39	8.79	2.86	22.42
10-20	1.50	8.90	2.72	22.18
20-30	1.50	8.81	2.67	21.95
30-40	1.59	8.69	2.65	21.73
40-50	1.61	8.85	2.67	21.96
50-60	1.57	8.67	2.53	21.32
60-70	1.62	8.82	2.43	21.51
70-80	1.60	8.63	2.27	20.87
80-90	1.62	8.80	2.30	21.05
90-100	1.59	8.64	2.10	20.42

EC_e_, electrical conductivity; SAR_e_, sodium adsorption rate.

### 2.2 Experimental design

**Figure 1:**
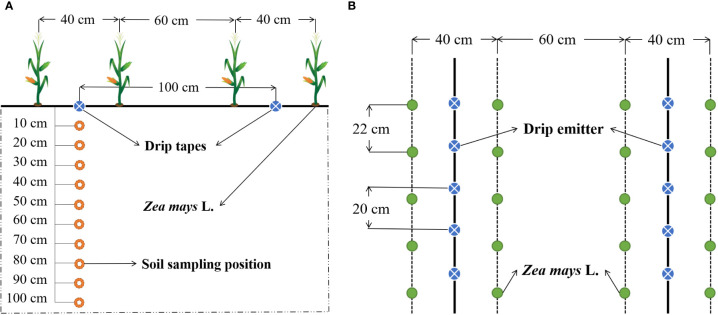
Treatment plot planting pattern and soil sampling sites. **(A)** Cross-section view; **(B)** Top view.

The study commenced on April 30^th^, 2022, with a trial period of 130 days. The test maize variety was Dajingjiu 26. Seeds were sown in parallel rows with wide (60 cm) and narrow (40 cm) between-row spacings and a planting density of 22 × 50 cm (**Figure 1**). The plants were irrigated using a drip irrigation system comprising solenoid valves, pressure meters, flow meters, screen filters, and fertilizer tanks. Drip irrigation tape was laid along the narrow rows with a drip head spacing of 20 cm and a drip head flow rate of 1.27 L h^-1^. As treatments, the effects of following four applications were assessed: (1) chemical fertilizer (CK); (2) humic acid with chemical fertilizer (HA); (3) carboxymethyl cellulose with chemical fertilizer (CMC); and (4) amino acids with chemical fertilizer (AA). Each treatment was performed in triplicate, giving a total of twelve 20 × 20 m^2^ randomly arranged plots.

Each plot was fertilized with the same total amounts of nutrients. N, P, and K were applied as 400 kg N ha^-1^, 200 kg P_2_O_5_ ha^-1^, and 400 kg K_2_O ha^-1^, respectively, of which 65% of the applied N was used as base fertilizer and 35% as a top-dressing. The water-soluble fertilizer (24–12–14) specifically formulated for maize drip irrigation was applied to the field along with the irrigation water via the drip irrigation system, according to established fertilizer demand during the maize growth period, and the irrigation quota used for all treatments was 450 mm. HA was purchased from Hongxinyuan Chemical Co., Ltd (black-brown powder solid, pH 4.3, total N ≤ 3.0%, total P ≤ 1%, and moisture ≤ 2.5%) and spread at 1500 kg per hectare prior to planting. CMC was purchased from Asher Chemical Co., Ltd (white flocculent fiber powder solid, pH 7–8, and moisture ≤ 1.5%) and spread at 200 kg per hectare prior to planting. AA was purchased from Chaoyixing Chemical Co., Ltd (yellowish-brown powder solid, pH 3.5, total N ≤ 15%, total P ≤ 0.5%, total K ≤ 3%, and moisture ≤ 3.0%) and spread at 1500 kg per hectare prior to planting. Rotary tillage was carried out immediately after spraying to a depth of 20–25 cm. Depending on the nutrient content of the soil amendment, the amount of fertilizer applied to HA, CMC, and AA treatment plots was adjusted to ensure that the amounts of nutrient applied were the same in each case, as shown in **Table 2**.

**Table 2:** Fertilization and irrigation during maize growing seasons .

Treatment	Chemical Fertilizer			HA	CMC	AA	Irrigation amount
	N	P_2_O_5_	K_2_O				
	(kg ha^−1^)			(kg ha^−1^)	(kg ha^−1^)	(kg ha^−1^)	(mm)
CK	400	200	400	0	0	0	450
HA	355	131	400	1500	0	0	450
CMC	400	200	400	0	200	0	450
AA	175	166	292	0	0	1500	450

CK is chemical fertilizer, HA is humic acid with chemical fertilizer, CMC is potassium carboxymethyl cellulose with chemical fertilizer, AA is amino acid with chemical fertilizer, the same below.

### 2.3 Soil sample collection and analysis

Soil samples were collected under the drip emitters in each plot using an auger (4.0 cm in diameter and 15 cm in height) and a cutting ring (10 cm in diameter and 10 cm in height) from the end of the milk stage (R3) to the beginning of the dough stage (R4) of maize growth. Samples were collected at depths of 10, 20, 30, 40, 50, 60, 70, 80, 90, and 100 cm, as shown in **Figure 1A**. In total, we collected 240 soil samples (120 auger samples and 120 cutting ring samples). Having removed surface organic impurities and fine roots from fresh auger-collected samples, a portion of each sample was oven dried to determine the soil moisture content. The remainder of the samples was naturally dried, passed through a 1-mm sieve, and used to produce a saturated soil slurry extract using a standard method (Robbins and Wiegand, 1990). The pH and electrical conductivity (EC_e_) were measured using a pH meter (PHS-3C, REX) and conductivity meter (DDS-12A, REX), respectively. Na^+^, Ca^2+^, and Mg^2+^ were measured using an inductively coupled plasma optical emission spectrometer (Optima 5300DV) and the sodium adsorption rate (*SAR_e_*) was calculated as follows:


(1) $${SAR}_{e}=\frac{{Na}^{+}}{{{[(Mg}^{2+}{+Ca}^{2+})/2]}^{0.5}}$$

where the concentration of each cation is in mmol L^-1^.

The soil desalination rate (*SDR*, %), used to characterize the desalination process, was calculated as follows:


(2) $$SDR=\frac{E_{0}{-E}_{\text{i}}}{E_{0}}\times 100\%$$

where E_0_ is the initial soil EC_e_ (dS m^-1^) and E_i_ is the soil EC_e_ for each soil layer at different stages (dS m^-1^).

The AN, AP, AK, and OC contents in the remaining soil samples were determined using the alkali-hydrolysis diffusion method, molybdenum-antimony resistance colorimetry, flame photometry, and potassium dichromate volumetric method, respectively (Pansu and Gautheyrou, 2006). The soil samples collected using a cutting ring were naturally air-dried to the plastic limit of the soil (water content of approx. 10%–20%), gently broken along the natural fracture surface of the soil samples, and water-stable aggregate were separated by wet sieving to obtain macro-aggregates (>2 mm), small aggregates (0.25–2 mm), micro-aggregates (0.053–0.25 mm), and silt and clay (SC, <0.053 mm) (Elliott, 1986).

### 2.4 Plant sample collection and analysis

At the end of the milk stage (R3) to the beginning of the dough stage (R4) of maize growth, three maize plants were randomly selected from the inner crop rows of each plot and their plant height and stem diameter were measured. Meanwhile, the net photosynthesis rate (Pn), intercellular CO_2_ concentration (Ci), transpiration rate (Tr), and stomatal conductance (Gs) of maize leaves were determined using the GFS-3000 gas-exchange and fluorescence system (Heinz Walz GmbH, Effeltrich, Germany). For each treatment, nine maize plants were collected (36 plants in total). Following on-site measurement, entire plants were immediately transported to the laboratory and dried to a constant weight at 70°C, from which we calculated aboveground biomass and grain yield. Using the values obtained for aboveground dry matter and grain yield, the harvest index (%) of maize silage was calculated using the following formula:


(3) N=Y/M

where *Y* is the grain yield (kg ha^-1^) and *M* is the aboveground dry matter mass per unit area (kg ha^-1^).

Water-use efficiency (*WUE*, kg ha^-1^ mm^-1^) during the maize growing season was calculated using the following equation (Zhang et al., 2019):


(4) WUE=Y/ET

where *Y* is the grain yield of maize (kg ha^-1^) and *ET* is the evapotranspiration during the maize growth period (mm).

The soil water balance was calculated as follows:


(5) ET=I+P+ΔS+G-R-L-E

where *ET* is the evapotranspiration (mm) during the growth of maize, *I* is the irrigation amount (mm), *P* is the total precipitation (mm) collected using a field rain gauge, *ΔS* is the change in soil water storage (mm) estimated using the space-weighted mean method, *G* is the contribution of groundwater (mm), *R* is the surface runoff (mm), *L* is the amount of underground leakage (mm) calculated using a soil leakage monitor, and *E* is the amount of surface water evaporation (mm) monitored using a micro-lysimeter evaporator. As the terrain of the experimental area is flat, the average depth of maize roots was considerably greater than the average depth of groundwater, and consequently, *G* and *R* could be ignored in this study.

### 2.5 Statistical analyses

All data were recorded and classified using Microsoft Office Excel 2016, and figures were prepared using Origin 2022 (Origin Lab Co., Northampton, MA, USA). SPSS 19.0 (IBM Co., Armonk, NY, USA) was used perform one-way ANOVA, and Tukey’s honestly significant difference test was used to compare the differences between treatments. Structural equation model was created by SPSS 19.0 for data input and Amos (IBM Co., Armonk, NY, USA) to build. The significance level was *P* < 0.05, and all data are presented as the means of three replicates.

## 3 Results

### 3.1 Soil moisture, EC_e_, pH, and SAR_e_

**Figure 2:**
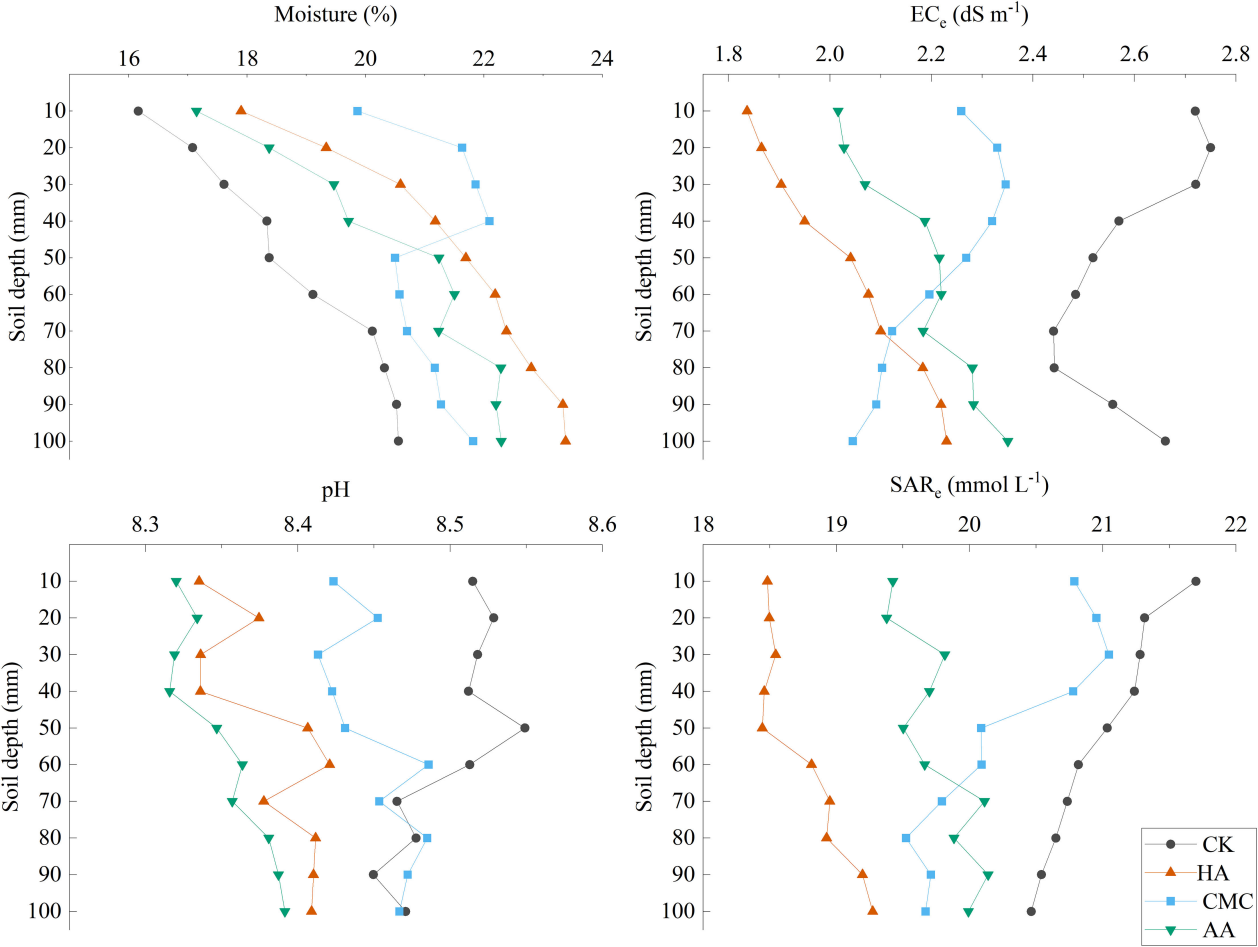
Spatial distribution of soil moisture, EC_e_, pH and SAR_e_.

**Figure 2** presents the variation characteristics of the soil moisture of different soil layers in response to the application of soil amendments, indicating that amendment had t significant effects on altering the infiltration characteristics of soil moisture. Overall, the soil moisture showed a trend of gradual increase with increasing soil depth, and the application of amendments resulted in significant increases in soil moisture content in the 0–40 cm and 40–100 cm soil layers. The water content of each treatment in the 0-40 cm soil layer showed CMC>HA>AA>CK, and the water content of each treatment in the 40-100 cm soil layer showed HA>AA>CMC>CK, which showed that CMC significantly increased the water content of the 0-40 cm soil layer in the CMC-treated soil due to its water absorption properties. Moreover, significant differences in the EC_e_ values of the different soil layers were recorded for each treatment. The application of amendments promoted a significant reduction in soil EC_e_, with values decreasing by different percentages in the HA-, CMC-, and AA-amended soils compared with the CK treatment. The lowest soil EC_e_ values were observed in the 0-50 cm soil layer for HA treatment, followed by AA treatment, CMC, and CK. In the 60-100 cm soil layer, soil EC_e_ values were lowest in CMC treatment, followed by HA treatment and AA treatment, and highest in CK. Similarly, the application of amendments resulted in a reduction in soil pH of the 0–60 cm soil layer compared with the CK-treated soil. Moreover, the trend in soil SAR_e_ was similar to that of soil EC_e_, with gradual declines in values with increasing soil depth observed in the CMC- and CK-treated soils, whereas gradual increases in values with soil depth were detected in the HA- and AA-amended soils. The average SAR_e_ of soil in the 0-100 cm soil layer under different treatments showed that CK > CMC > AA > HA.

### 3.2 Soil desalination

The results presented in **Table 3** indicate that all amendments had a significant impact on soil salinity and desalination rates in different soil layers. Notably, the salinity levels in the 0-40 cm layer of soils treated with HA, CMC, and AA were substantially lower than those in CK-treated plots, with respective reductions of 11.53%–34.78%, 10.59%–24.97%, and 7.22%–28.30%. Moreover, the average desalination rates in the 0-50 cm soil layer were much higher in soils amended with HA, CMC, and AA, compared to the CK treatment. Specifically, the tillage layer (0–40 cm) of amended soils demonstrated higher desalination rates (32.68%, 15.46%, and 25.16% for HA, CMC, and AA treatments, respectively) than the CK treatment (18.45%). However, the desalination efficiency of all treatments declined with increasing soil depth, as soil density increased and porosity decreased. In fact, in the 70-100 cm soil layer, negative rates of desalination were recorded, with CK showing the lowest desalination efficiency at -11.35%. Therefore, amendments can effectively reduce soil salinity and enhance the desalination rate, particularly in the tillage layer.

**Table 3:** The salinity and desalination rate of different soil layers.

Soil layer	Treatment	Initial soil salinity	Soil salinity at maturity	Soil desalination rate
(cm)		(g kg^-1^)	(g kg^-1^)	(%)
0-10	CK	10.19aA	9.72aA	4.64aC
	HA	10.09aA	6.34cC	37.17aA
	CMC	10.24aA	7.93abcB	22.60aB
	AA	10.32aA	7.01bBC	32.05aAB
10-20	CK	9.96abA	9.84aA	1.25abC
	HA	9.66abA	6.44cC	33.32abA
	CMC	9.68abA	8.20aB	15.26abB
	AA	9.66abA	7.05bC	26.96abA
20-30	CK	9.86abcA	9.72aA	1.39aC
	HA	9.57abcA	6.59bcC	31.19abcA
	CMC	9.36bA	8.27aB	11.66abB
	AA	9.35bcA	7.21abBC	22.77abcAB
30-40	CK	9.42abcA	9.13aA	3.14aC
	HA	9.53abcA	6.76bcC	29.04abcA
	CMC	9.31bdA	8.16abB	12.33abB
	AA	9.44bcA	7.66abB	18.84abcB
40-50	CK	9.51abcA	8.93aA	5.78aB
	HA	9.50bcA	7.10abcB	25.04bcdA
	CMC	9.54bA	7.97abcAB	16.52abA
	AA	9.5bcA	7.76abAB	18.14abcA
50-60	CK	8.97bcdA	8.80aA	1.85aB
	HA	9.05cdA	7.23abB	20.07cdA
	CMC	8.89bcdA	7.69abcdB	13.46abA
	AA	8.98cdA	7.78abAB	13.37bcdA
60-70	CK	8.79cdA	8.63aA	1.72aA
	HA	8.67deA	7.32abB	15.53deA
	CMC	8.53cdA	7.41bcdAB	13.12abA
	AA	8.41deA	7.64abAB	9.11cdeA
70-80	CK	7.98deA	8.64aA	-9.14abA
	HA	7.91fA	7.64aA	3.40fgA
	CMC	7.62eA	7.34cdA	3.60bA
	AA	7.67fgA	8.01abA	-4.54efA
80-90	CK	8.08deA	9.08aA	-12.32abB
	HA	8.20efA	7.78aAB	5.19efAB
	CMC	8.13deA	7.29cdB	9.88abA
	AA	7.91efA	8.02abAB	-1.41deAB
90-100	CK	7.56eA	9.49aA	-25.68bC
	HA	7.25gA	7.82aBC	-7.82gAB
	CMC	7.41eA	7.12dC	3.90bA
	AA	7.04gA	8.28aB	-17.70fBC

Different lowercase letters in the same column indicate significant differences between the same treatments in different soil layers, and different capital letters in the same column indicate significant differences between the same soil layers in different treatments (P < 0.05).

### 3.3 Soil aggregate structure

**Figure 3:**
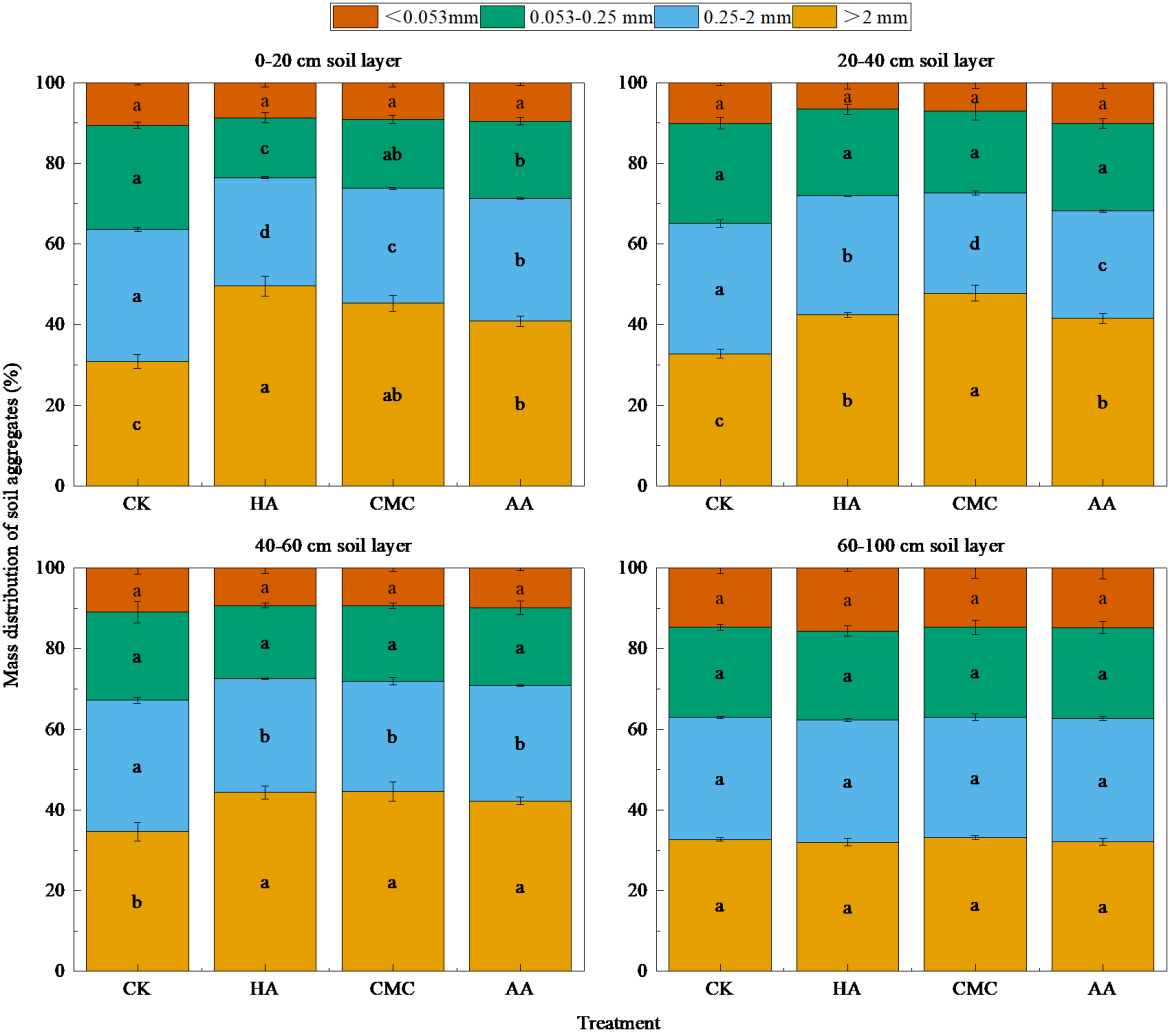
Structural characteristics of soil aggregates. Different lowercase letters for the same color indicate significant differences between different treatments for soil aggregates of the same particle size (*P* < 0.05).

To further investigate the effect of amendments on soil structure, undisturbed soil was divided into macro-aggregates (>2 mm), small aggregates (0.25–2 mm), micro-aggregates (0.053–0.25 mm), and SC (<0.053 mm) by wet screening for comparison and analysis. As shown in **Figure 3**, in each layer of the treated soils, macro-aggregates were the main structural components of soil, accounting for 30.85%–49.52% of the original soil mass, followed by small aggregates (26.95%–32.72%), micro-aggregates (14.83%–25.86%) and SC (8.70%–10.56%). In the 0–20 cm soil layer, compared with the CK treatment, we detected significant increases in the proportions of macro-aggregates of between 32.49% and 60.52% in the different amended soils, with the following trend: HA ≥ CMC > AA > CK. Contrastingly, there were reductions of 7.69%–21.41% and 35.03%–75.39% in the proportion of soil small-aggregates and micro-aggregates, respectively, in the order CK > AA ≥ CMC > HA. The application of amendments also increased the proportion of macro-aggregates in the 20–40 cm soil layer, with corresponding reductions in the proportions of small aggregates and micro-aggregates. Among the assessed treatments, the highest proportion of macro-aggregates (reaching 47.83%) was obtained in the CMC-amended soil. With increasing soil depth, there was a gradual reduction in the differences in soil aggregate structure, with no significant differences among the different treatments, although the proportion of macro-aggregates in the 40–60 cm soil layer was still significantly higher than that in the CK-treated soil by 21.90%–28.50%. In the 60–100 cm soil layer, there were no significant differences among treatments for any of the assessed aggregate types.

### 3.4 Soil available nutrients

AN: For all treatments, we observed a gradual reduction in alkali-hydrolyzable N with an increase in soil depth. Among the different treatments, the CK-treated soil was found to have the lowest soil alkali-hydrolyzable N content, followed by CMC, with AA- and HA-amended soil having the highest contents.

AP: Compared with the CK treatment, the contents of soil available P were significantly higher in all amended soils, although there were no significant differences among the different amendment treatments. With an increase in soil depth, there was a gradual reduction in the content of available P.

AK: Similar to the patterns observed for alkali-hydrolyzable N and AP, we detected a gradual reduction in soil AK with increasing soil depth. In the 20–60 cm soil layer of HA-, AA-, and CMC-amended soils contents were 21.63%–38.04%, 10.69%–19.30%, and 20.72%–38.92% higher compared with those in the CK-treated soil, respectively. In the 100 cm soil layer, AK contents in the HA- and AA-amended soils were 9.06%–32.55% and 13.14%–38.83% higher than those in soils treated with CMC and CK, respectively.

OC: There was a gradual reduction in the OC content of soil with increasing soil depth. In the 0–70 cm soil layer, soil amendments were found to promote significant increases in soil OC contents. In the 0–30 cm soil layer, compared with the CK-treated soil, carbon contents were 43.05%–49.80%, 14.80%–19.81%, and 27.39%–86.44% higher in the HA-, AA-, and CMC-amended soils respectively.

### 3.5 Maize water consumption

As shown in **Table 4**, soil water storage (Δ*S*) values ranged from 36.63 to 48.18 mm throughout the growing season, with the highest values being recorded in CMC-amended soil, followed by those in soils treated with HA, AA, and CK. Compared with the CK treatment, the application of amendments was found to promote significant reductions in underground leakage (*L*) and surface evaporation (*E*) of 23.24%–41.34% and 22.40%–40.49%, respectively, with CMC-amended soil being characterized by the lowest surface evaporation of 113.9 mm. It can be seen that in the CK-treated soil, the underground leakage and surface evaporation account for 33.76% of the applied irrigation water and rainfall, which is significantly higher than that of soil amended with HA (26.33%), CMC (20.07%), and AA (26.16%). It is noteworthy that the *ET* of CMC-treated soil (up to 543mm), was significantly higher than that of soils receiving the other treatments, but the *WUE* of this soil (20.56 mm) was significantly lower than that of other treatments, thereby indicating that whereas the water-retention properties of CMC improved soil water content, it was not conducive to the absorption and utilization of this soil water by maize. Otherwise, we detected no significant differences among the CK treatment, HA, and AA treatments with respect to *WUE*.

**Table 4:** Maize (*Zea mays* L.) evapotranspiration components and water use efficiency.

Treatment	*I*	*P*	Δ*S*	*L*	*E*	(*L*+*E*)/(*I*+*P*)	*ET*	*WUE*
	(mm)	(mm)	(mm)	(mm)	(mm)	(%)	(mm)	(kg ha^−1^ mm^−1^)
CK	450	169	36.63b	17.67a	191.3a	33.76a	446.6c	23.43a
HA	450	169	43.27a	13.57b	149.4b	26.33b	499.3b	24.81a
CMC	450	169	48.18a	10.37b	113.9c	20.07c	543.0a	20.56b
AA	450	169	41.91ab	13.50b	148.5b	26.16b	499.0b	24.50a

I is the amount of irrigation, P is the precipitation during the maize growing season, ΔS is the change in soil water storage in the 0-100 cm soil layer, L is the subsurface seepage, E is the evaporation of surface water, ET is the evapotranspiration during maize growth, and WUE is the water use efficiency. Different lowercase letters in the same column indicate significant differences between different treatments at P < 0.05.

### 3.6 Maize photosynthetic parameters

Compared to the CK treatment, the application of HA and AA resulted in significant increases in Pn, with AA exhibiting the highest value of 55.74 μmol m^-2^ s^-1^ (**Table 5**). Additionally, HA treatments led to elevated Ci levels, indicating an increase in the efficiency of CO_2_ utilization. Meanwhile, CMC did not significantly affect the photosynthetic parameters, suggesting its limited impact on the overall physiological performance of maize leaves. Furthermore, all treatments showed a similar Tr and Gs, suggesting that the different treatments did not significantly impact water loss or stomatal conductance. In summary, the application of HA and AA to maize plants led to significant improvements in photosynthetic parameters, with AA showing the most pronounced effects. Meanwhile, CMC did not significantly influence the photosynthetic performance of maize leaves.

**Table 5:** Photosynthetic parameters in maize (*Zea mays* L.) leaves.

Treatments	Pn	Ci	Tr	Gs
	(umol m^-2^ s^-1^)	(ppm)	(mmol m^-2^ s^-1^)	(mmol m^-2^ s^-1^)
CK	48.92b	56.66ab	5.87a	247.6a
HA	55.12a	89.07a	7.51a	323.1a
CMC	50.56ab	44.85b	7.01a	260.2a
AA	55.74a	77.02ab	7.67a	313.6a

Pn is net photosynthetic rate, Ci is intercellular CO_2_ concentration, Tr is transpiration rate, Gs is stomatal conductance. Different lowercase letters in the same column indicate significant differences between different treatments at P < 0.05.

### 3.7 Maize yield and quality

As shown in **Table 6**, there were no significant effects of soil amendment on the height or stalk diameter of maize plants. Contrastingly, however, we detected significant differences among treatments with respect to aboveground biomass, grain yield. and nutrient index. Compared with the CK treatment, there was a significant increase of 2.53%-9.47% in the aboveground biomass of maize grown in HA-amended soil, although we detected no significant differences in this regard among the different amendment treatments. Compared with the CK treatment, we detected significantly higher (by 16.81%–18.32%) grain yields for maize grown in the HA- and AA-amended soils, reaching 12226-12384 kg ha^-1^, although these values did not differ significantly from those obtained for maize cultivated in CMC-amended soil. Values obtained for the harvest index of maize grain yield and aboveground biomass indicated that the application of HA and AA contributed to a significant improvement in the nutritional index of silage maize, reaching 20.53%–20.61%, although we detected no significant difference between CMC amendment and any other treatment in this regard. In conclusion, these findings revealed some differences in the effectiveness of different soil amendments in fertilization, soil improvement and crop growth promotion, where HA and CMC treatments had excellent yield increasing effects by increasing above-ground biomass and seed yield by 4.82-6.14% and 16.81-18.32%, respectively, compared to CK treatment.

**Table 6:** Maize (*Zea mays* L.) morphology and yield.

Treatment	Plant height	Stem diameter	Aboveground biomass	Grain yield	Harvest index	Aboveground biomass increase	Grain yield increase
	(cm)	(mm)	(kg·ha^-1^)	(kg·ha^-1^)	(%)	(%)	(%)
CK	284.9a	19.35a	45248b	10466c	23.14b	–	–
HA	309.8a	20.21a	48026a	12384a	25.79a	6.14	18.32
CMC	285.5a	19.51a	46374ab	11158bc	24.06ab	2.49	6.60
AA	289.3a	19.70a	47430ab	12226ab	25.77a	4.82	16.81

Different lowercase letters in the same column indicate significant differences between different treatments at P < 0.05.

The results obtained from this study, presented in **Table 7**, demonstrate that the application of CMC, HA, and AA did not significantly alter the soluble sugar, crude fat, and starch content of maize grain. However, the application of HA and AA significantly enhanced the crude protein content of maize grain compared to the control group (CK) and CMC. The increase in crude protein content of maize grain can be attributed to the fact that the active ingredients in HA and AA soil conditioners provided a favorable environment for the development of beneficial soil microorganisms, thus promoting the mineralization of organic matter, increasing the nutrient utilization of the crop and improving protein synthesis in maize grain.

**Table 7:** Maize (*Zea mays* L.) grain quality.

Treatments	Soluble Sugar	Crude Protein	Crude Fat	Starch
	(%)	(%)	(%)	(%)
CK	2.64a	8.24bc	3.10a	67.32a
HA	2.79a	8.50ab	3.21a	68.02a
CMC	2.70a	8.16c	3.09a	68.74a
AA	2.77a	8.56a	3.22a	68.77a

Different lowercase letters in the same column indicate significant differences between different treatments at P < 0.05.

### 3.8 Correlation analysis

**Figure 4:**
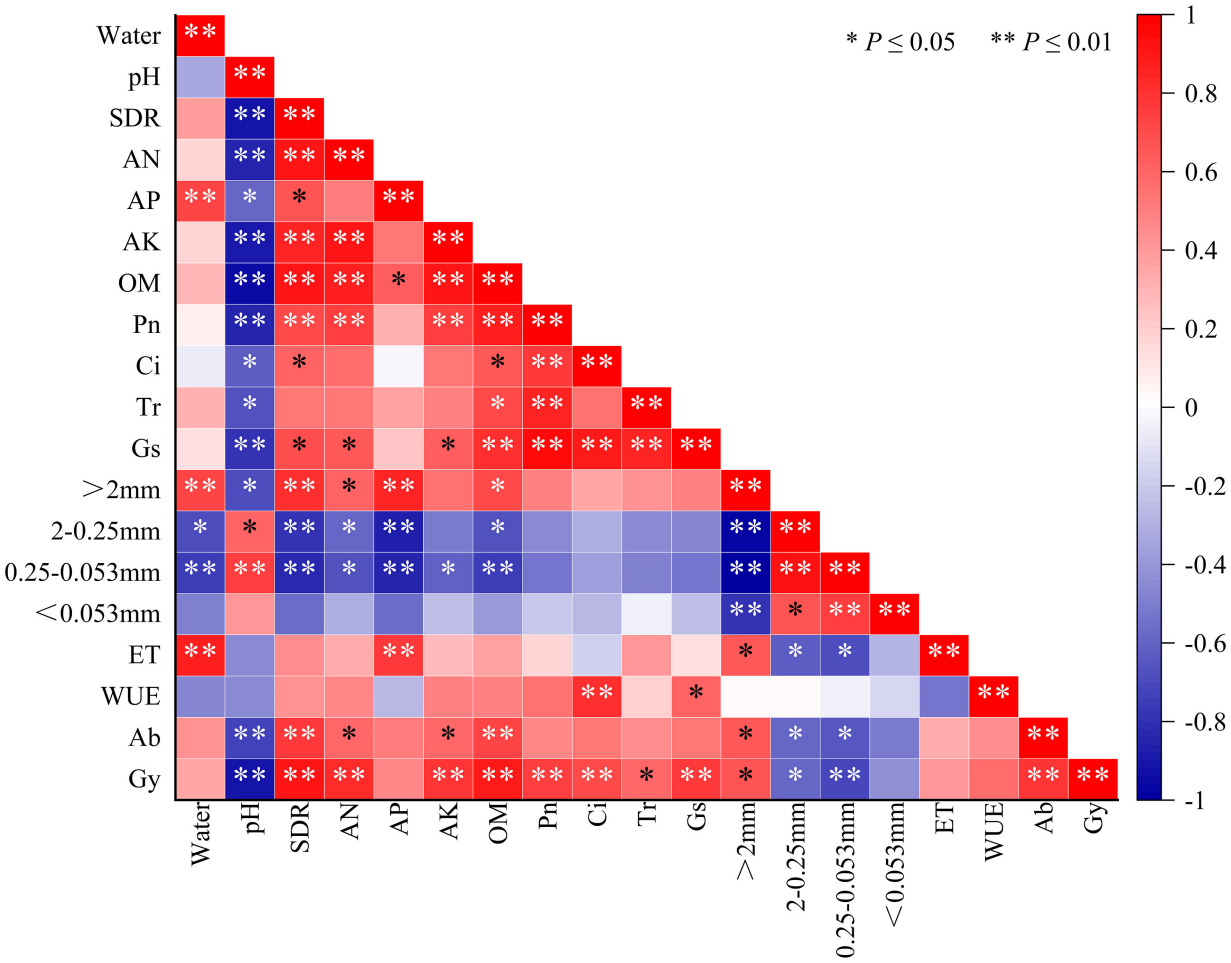
Correlation analysis of maize growth and soil physical and chemical properties. Water: soil water content; pH: soil pH value; SDR: soil desalination rate; AN: alkali-hydrolyzable nitrogen; AP: available phosphorus; AK: available potassium; OM: organic carbon; Pn: net photosynthesis rate; Ci: intercellular CO_2_ concentration; Tr: transpiration rate; Gs: stomatal conductance; >2 mm: macro-aggregates; 2-0.25 mm: small aggregates; 0.25-0.053 mm: micro-aggregates; <0.053 mm: silt and clay; ET: evapotranspiration; WUE: water-use efficiency; Ab: aboveground dry matter; Gy: grain yield.

A correlation heatmap is a powerful visualization tool that serves to depict the interrelationships between different variables. Through a correlation analysis of soil physicochemical properties and maize growth indicators (**Figure 4**), we discovered that the pH level exhibited negative correlations with various soil physicochemical properties and maize growth indicators. On the other hand, soil desalination rate and effective nutrient content were positively correlated with net photosynthesis parameters, percentage of soil macro-aggregates (>0.02 mm), aboveground biomass and maize yield. These interesting findings suggest that the application of organic amendments may be able to promote maize growth, photosynthetic parameters, and ultimately maize yield by promoting soil macro-agglomerate formation, decreasing soil salinity, and increasing soil effective nutrient content.

**Figure 5:**
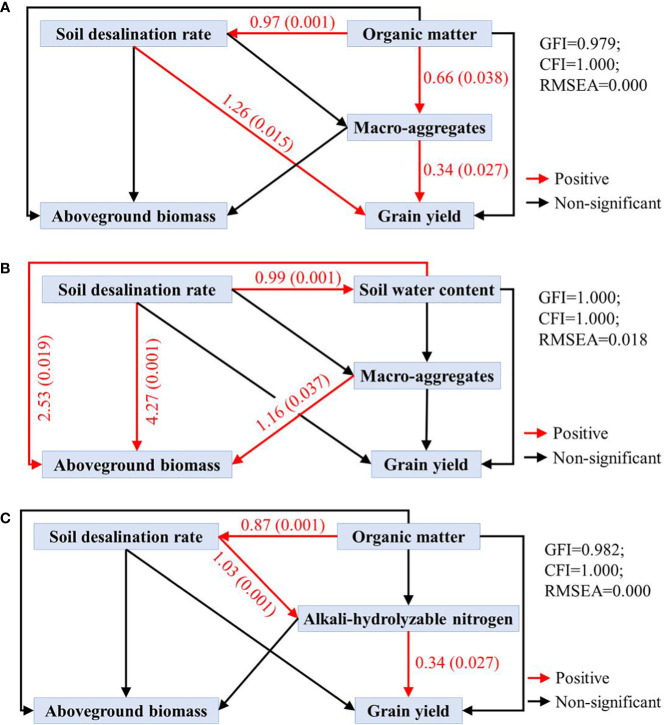
SEM of HA, CMC and AA. **(A)** denotes the SEM of HA; **(B)** denotes the SEM of CMC; **(C)** denotes the SEM of AA. The red line denotes a positive relationship and the black line denotes a non-significant relationship. Numbers next to paths are normalized path coefficients and corresponding P values are in parentheses.

Structural equation model (SEM) combines validated factor analysis and path analysis to simultaneously consider complex causal relationships between multiple independent and dependent variables. As shown in**Figure 5**, maize grain yield under HA treatment was directly affected by soil desalination rate (p=0.015) and soil macro-aggregate percentage (p=0.27), and indirectly affected by soil organic matter content. Maize aboveground biomass under CMC treatment was directly affected by soil desalination rate (p=0.001), soil water content (p=0.019) and soil macro-aggregate percentage (p=0.037). Maize grain yield under AA treatment was directly affected by soil alkali-hydrolyzable N (p=0.027) and indirectly by soil desalination rate and soil organic matter.

## 4 Discussion

### 4.1 Soil desalination process

Soil amendments, such as HA, wood vinegar, cow manure composite, and biochar, have been reported to improve the physicochemical properties of saline-alkali soil, reducing salinity and pH and alleviating salt stress (Li et al., 2022; Pan et al., 2022). Our study investigated the variability characteristics of water and salt distribution in saline-alkali soil in response to the application of different soil amendments. The results revealed significant differences in the water contents of different soil layers treated with various soil amendments, with the water content gradually increasing with increasing soil depth (**Figure 2**). HA and biochar application, known to increase soil water content while reducing salinity and pH (Khaled and Fawy, 2011; Sun et al., 2020), align with our observations, indicating the potential of these amendments to enhance soil water content in different soil layers. Organic matter-rich amendments, such as HA and AA, increased soil water content due to increased soil aggregation and water retention capacity, reducing water loss through evaporation and drainage, mitigating salt stress (Pettit, 2004). In addition, the lower pH organic acids in amendments such as HA and AA help to neutralize certain free OH^-^ ions, thereby improving the soil microenvironment. HA and AA both have the potential to improve soil water retention and enhance soil structure. HA’s “sponge-like” structure enables it to absorb and retain water, leading to increased soil water content and reduced evaporation. Moreover, HA promotes the formation of stable soil aggregates, which can improve soil porosity and water infiltration (Lima et al., 2009; Smebye et al., 2016). On the other hand, AA can enhance soil aggregation through microbial interactions and organic matter decomposition, but its direct water absorption capacity is relatively limited compared to HA. Therefore, in situations where improving water retention and soil structure are critical, HA may be preferred due to its superior water-absorption capabilities and aggregate-promoting properties.

However, we observed unique effects of CMC on soil water and salt distribution. CMC has a stronger water absorption potential (Nie et al., 2004; Kim et al., 2021), which increased water and salt content in shallow soil (0–40 cm) but had limited effects on water retention and desalination in deeper soil layers (60–100 cm) (**Figure 2**). This difference may be attributed to the hydrophilicity of CMC, which adsorbs water molecules to form hydrogels indirectly promoting the coagulation of soil particles, leading to the blockage of some soil pores and a decrease in infiltration rate, which reduces the amount of water lost by evaporation and drainage, thus increasing the soil water content. Whereas, soil water is a carrier for the movement of salts, which leads to the accumulation of salts in the 20-40 cm soil layer, which may create unfavorable soil conditions for normal crop growth, which may affect crop growth (Olad et al., 2018). In other words, the increased water retention properties of the soil, while reducing the upward and downward movement of water and impeding the movement of salts, also immobilizes to some extent the salts that were already present in the surface soil. Therefore, although improving water retention in saline soils is an important goal in combating the adverse effects of high salinity on crop growth, it is necessary to carefully consider the use of CMC as a soil amendment in order to avoid potential trade-offs between increased water retention and salt accumulation.

### 4.2 Soil structure and nutrients

Soil aggregates are formed by the cementation and aggregation of soil primary particles, which can reflect the degree of the structural aggregation and fertility properties, of soil, and is considered as a material basis for the formation of soil aggregate structure (Peth et al., 2008). In our study, we found that the application of amendments contributed to a significant increase in the proportion of macro-aggregates and a reduction in the proportion of small aggregates in the 0–60 cm soil layer (**Figure 3**). These effects are assumed to be associated with the decomposition of organic amendment material to organic cementitious substances following application, the viscosity and cementation of which can promote the formation of large-diameter aggregates, thereby reducing the content of small aggregates. In addition, we found that in the 0–20 cm soil layer, the promotion effect of HA on the formation of large soil aggregates was significantly superior to that of the other assessed amendments, in that the unique “sponge-like” structure of HA can contribute to enhancing soil porosity due to water absorption and expansion (Elliott, 1986). Moreover, during the infiltration process, the colloidal properties of HA can promote the formation of stable aggregates from loose particles (Zhao et al., 2022), thereby increasing the effective water storage space, and thus also increasing the soil water content.

**Figure 6:**
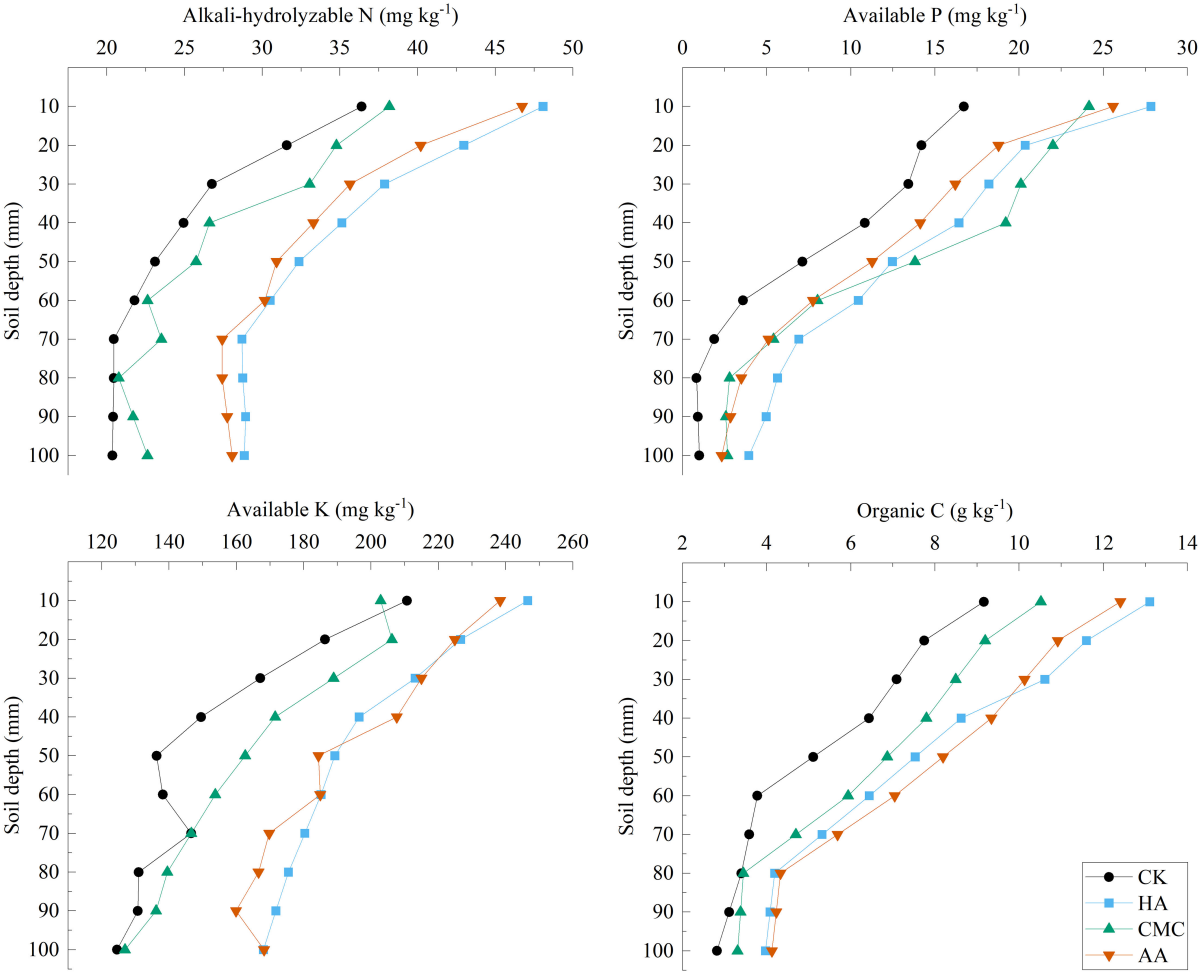
Spatial distribution of soil available nutrients.

In this study, we observed that compared to the CK treatment, the addition of HA and AA increased the levels of soil OC (23.36%–86.44%), alkali-hydrolyzable N (27.27%–41.80%), AP (20.77%–576.8%), and AK (13.14%–38.92%) to varying degrees (**Figure 6**). It can be speculated that these effects are attributable to the high organic matter contents of these amendments, which contribute to a direct increase in the effective nutrient content of the soil, with subsequent microbial mineralization during the latter stages of maize development (Masunga et al., 2016; Siedt et al., 2021a). In contrast, the CK treatment only supplies inorganic fertilizers, and most of the nutrients may be lost with the infiltration of drip irrigation water. In addition, the use of organic amendments could promote the formation of large agglomerates that sequester soil nutrients, thus preventing microbial decomposition and leaching (Bronick and Lal, 2005; Bailey et al., 2019), ultimately improving the overall efficiency of soil nutrient sources, which may also be one of the main reasons for increased soil fertility. Therefore, both HA and AA are organic-based soil improvers that can enhance soil organic matter content and improve nutrient availability. However, HA, being a complex mixture of humic substances, has a higher cation exchange capacity (CEC) and more diverse functional groups (Jing et al., 2020). These properties allow HA to bind and chelate essential nutrients, such as potassium, calcium, and magnesium, making them more available for plant uptake. Additionally, HA can stabilize nutrients in the soil, reducing nutrient leaching and improving nutrient use efficiency (Pettit, 2004). While AA can also contribute to nutrient availability through microbial activity and organic matter decomposition, its impact on nutrient retention and stabilization may not be as pronounced as HA.

### 4.3 Crop responses

We investigated the impact of soil amendments on various aspects of maize growth, nutrient uptake, physiological indicators, grain yield, and nutritional quality. In our study, we observed that the application of soil amendments significantly reduced surface evaporation and underground leaching of farmland water while increasing maize evapotranspiration (**Table 4**). Consequently, the proportion of water loss in both categories relative to the overall water input decreased, resulting in an improvement in water use efficiency. This enhancement was attributed to the ability of organic matter present in the soil modifiers to combine with clay particles, leading to more stable soil aggregates, increased soil porosity, and water permeability, consequently reducing water loss (Atkinson et al., 2010). Furthermore, the organic matter promoted the growth and metabolism of soil microorganisms, increasing soil organic matter and biodiversity, and further decreasing the leaching of soil water while improving the soil’s water retention capacity (Siedt et al., 2021a). It is important to clarify that despite enhancing soil fertility, HA and AA do not directly improve crop water use efficiency as they lack the ability to absorb water, thus not influencing water absorption and utilization of crops (**Table 4**). Notably, the water-use efficiency of CMC-amended soil was the lowest among the assessed amendment treatments, with a value of only 20.56 kg ha^-1^ mm^-1^ (**Table 4**). This observation could be attributed to the fact that although CMC enhances water retention in the rhizosphere, the water present is in a free soluble state in the soil, which is not conducive to plant absorption and utilization. Thus, this could be one of the factors leading to the low maize grain yield observed in CMC-amended soil.

Our study showed that the application of HA and AA significantly increased Pn and Ci in maize plants (**Table 5**). This effect is attributed to the enhanced nutrient uptake and utilization by crops, as well as the enhanced diversity and number of soil microbial communities. These factors promote the metabolic activity and nutrient cycling of microorganisms, thus supporting plant photosynthesis and carbon cycling. The maize variety grown in this study was suitable for silage production and were therefore harvested at a relatively early growth stage and had a relatively low harvest index compared to grain maize varieties. Harvest index is the ratio of grain yield to aboveground dry matter mass of a crop and can also characterize the nutritional quality of maize for silage (Lima et al., 2022). Although no significant differences in maize plant heights or stem diameters were observed in soils treated with any of the assessed amendments, the application of amendments contributed significantly to the enhancement of aboveground biomass and grain yield, concurrently improving nutritional quality and economic value (**Table 6**). Notably, HA amendment demonstrated the most pronounced effect on grain yield, yielding up to 12,384 kg ha^-1^, a remarkable 13.54%–22.71% higher than those grown in CK-treated soil. Moreover, the application of AA led to a significant increase in protein content in maize grain (**Table 7**). This can be attributed to AA being a component of plant protein, and their application increases soil nitrogen sources, resulting in an elevated crude protein content.

Significantly, the compatibility of HA, CMC, and AA as soil conditioners with slow-release fertilizers, animal manure, and chemical fertilizers represents a pivotal consideration in sustainable agricultural practices. When combined with animal manure or slow-release fertilizers, HA and AA have demonstrated remarkable proficiency in stimulating nutrient release and catalyzing the decomposition of organic matter. This expedites nutrient mineralization, ensuring a sustained and consistent supply of vital nutrients essential for crop growth (Kandil et al., 2016; Saudy et al., 2020). Notably, as organic-based soil enhancers, HA and AA possess the inherent capacity to synergistically complement slow-release fertilizers, amplifying nutrient retention and availability within the soil matrix. Conversely, CMC plays a pivotal role in preserving soil moisture and prolonging the release of nutrients from slow-release fertilizers, thereby facilitating the efficient assimilation of nutrients by plants (Davidson et al., 2013; Guilherme et al., 2015). Additionally, the presence of abundant soil moisture fosters heightened microbial activity, thereby promoting the mineralization of organic matter present in animal manure, ultimately enriching the soil’s effective nutrient reservoir. In summation, the harmonious compatibility of distinct soil enhancers, such as HA, CMC, and AA, with diverse fertilizer types stands as an indispensable consideration when implementing these treatments in agricultural practices. While organic-based soil enhancers, such as HA and AA, generally exhibit propitious interactions with slow-release fertilizers and manure, prudent caution is warranted when deploying them in conjunction with certain chemical fertilizers. Thus, future studies should prioritize assessing these intricate interactions and optimizing the judicious integration of soil enhancers with diverse fertilizers to maximize their cumulative advantages in elevating soil fertility and augmenting crop productivity.

Correlation analysis and structural equation modeling (**Figure 5**) revealed that the application of HA can enhance maize grain yield by increasing soil organic matter, reducing soil salinity, and improving soil desalination efficiency. CMC application can elevate aboveground biomass by enhancing soil desalination efficiency, increasing soil water content, and promoting the formation of soil aggregates. Meanwhile, the application of AA can boost maize grain yield by augmenting soil organic matter, promoting soil desalination, and increasing soil available nitrogen content. These findings underscore the distinct mechanisms behind the yield-enhancing effects of HA, CMC, and AA in saline-alkali soil. HA achieves yield improvement through the enhancement of organic matter and facilitation of soil aggregate formation, CMC relies on its excellent water retention properties to increase soil water content and promote salt leaching, while AA enhances yield through increased soil organic matter and available nitrogen content. Additionally, it is worth noting that the three amendments exhibit significant cost differences, with HA priced at $0.18 per kilogram, CMC at $1.54 per kilogram, and AA at $0.17 per kilogram. Based on the experimental field application rates, the cost of amending one hectare of saline-alkali soil would be $269 (HA), $307 (CMC), and $261 (AA), respectively. Therefore, considering the distinct characteristics of different soil amendments, local soil conditions, and economic factors, the optimal approach would be to employ a composite of these amendments to achieve the best results. This direction can be pursued in future research endeavors.

One of the main findings of this study was the positive effects of HA treatments on soil properties, such as reduced soil salinity, improved soil aggregate stability promoting better water infiltration and retention, and improved water use efficiency. These improvements in soil properties are essential for sustainable development of saline soils as they help create a more favorable environment for plant growth and crop productivity. Another important finding of this study was that maize yield and nutrient quality were significantly improved by the application of HA. The HA promoted increased nutrient effectiveness and nutrient use efficiency in the soil, which positively affected crop growth and development. In the Yellow River Irrigation District, the use of organic soil amendments, such as HA and AA, provides a sustainable alternative to conventional chemical fertilizers. Chemical fertilizers are commonly used to improve soil fertility; however, they often lead to soil degradation, nutrient imbalance, and environmental pollution (Bisht and Chauhan, 2020). In contrast, HA and AA treatments promote soil health and fertility by adding organic matter and enhancing nutrient cycling, which contributes to long-term sustainable agricultural practices. In conclusion, this study demonstrated the great potential of HA as a soil amendment to improve soil properties, enhance crop yield and quality, and promote sustainable agricultural practices in saline and alkaline soils of the Yellow River Irrigation District. The adoption of these organic soil treatments provides a viable and environmentally friendly alternative to conventional fertilizers and contributes to the sustainable development and exploitation of saline soils.

## 5 Conclusion

Our research highlights the efficacy of humic acid, carboxymethyl cellulose, and amino acids as soil amendments in ameliorating water infiltration characteristics of saline soils, mitigating soil salinization, and enhancing soil nutrient availability and maize yield. Despite their distinct mechanisms of yield enhancement, humic acid emerges as the most promising soil amendment, recommended for treating local saline soils. However, given the intricate and multifaceted nature of soil environments, the application of a single soil amendment may not suffice to achieve optimal outcomes concerning water conservation, salt suppression, nutrient availability, and sustained crop productivity. Moreover, considering cost implications, it becomes imperative to delve into the investigation of various combinations of amendments and their underlying mechanisms for future studies.

## Data availability statement

The original contributions presented in the study are included in the article/supplementary material. Further inquiries can be directed to the corresponding author.

## Author contributions

YW and RW contributed to the design of the study, and YW wrote the first draft of the manuscript. MG, HC and YC conducted field sampling, laboratory analysis and statistical analysis. LW wrote sections of the manuscript. All authors contributed to the article and approved the submitted version.

## References

[B1] Atkinson C. J. Fitzgerald J. D. Hipps N. A. (2010). Potential mechanisms for achieving agricultural benefits from biochar application to temperate soils: a review. Plant Soil 337, 1–18. doi: 10.1007/s11104-010-0464-5

[B2] Bailey V. L. Pries C. H. Lajtha K. (2019). What do we know about soil carbon destabilization? Environ. Res. Lett. 14 (8), 083004. doi: 10.1088/1748-9326/ab2c11

[B3] Bisht N. Chauhan P. S. (2020). Excessive and disproportionate use of chemicals cause soil contamination and nutritional stress. Soil contamination-threats Sustain. solutions, 1–10. doi: 10.5772/intechopen.94593

[B4] Bronick C. J. Lal R. (2005). Soil structure and management: a review. Geoderma 124 (1-2), 3–22. doi: 10.1016/j.geoderma.2004.03.005

[B5] D'hose T. Debode J. De Tender C. Ruysschaert G. Vandecasteele B. (2020). Has compost with biochar applied during the process added value over biochar or compost for increasing soil quality in an arable cropping system? Appl. Soil Ecol. 156, 103706. doi: 10.1016/j.apsoil.2020.103706

[B6] Davidson D. W. Verma M. S. Gu F. X. (2013). Controlled root targeted delivery of fertilizer using an ionically crosslinked carboxymethyl cellulose hydrogel matrix. SpringerPlus 2 (1), 1–9. doi: 10.1186/2193-1801-2-318 23961392 PMC3724987

[B7] De Castro T. A. V. T. Berbara R. L. L. Tavares O. C. H. Da Graca Mello D. F. Pereira E. G. De Souza C. D. C. B.. (2021). Humic acids induce a eustress state via photosynthesis and nitrogen metabolism leading to a root growth improvement in rice plants. Plant Physiol. Biochem. 162, 171–184. doi: 10.1016/j.plaphy.2021.02.043 33684776

[B8] Elliott E. (1986). Aggregate structure and carbon, nitrogen, and phosphorus in native and cultivated soils. Soil Sci. Soc. America J. 50 (3), 627–633. doi: 10.2136/sssaj1986.03615995005000030017x

[B9] Fu X. Wu X. Wang H. Chen Y. Wang R. Wang Y. (2023). Effects of fertigation with carboxymethyl cellulose potassium on water conservation, salt suppression, and maize growth in salt-affected soil. Agric. Water Manage. 287, 108436. doi: 10.1016/j.agwat.2023.108436

[B10] Guilherme M. R. Aouada F. A. Fajardo A. R. Martins A. F. Paulino A. T. Davi M. F.. (2015). Superabsorbent hydrogels based on polysaccharides for application in agriculture as soil conditioner and nutrient carrier: A review. Eur. Polymer J. 72, 365–385. doi: 10.1016/j.eurpolymj.2015.04.017

[B11] Jing J. Zhang S. Yuan L. Li Y. Lin Z. Xiong Q.. (2020). Combining humic acid with phosphate fertilizer affects humic acid structure and its stimulating efficacy on the growth and nutrient uptake of maize seedlings. Sci. Rep. 10 (1), 17502. doi: 10.1038/s41598-020-74349-6 33060730 PMC7562911

[B12] Kandil A. Sharief A. Seadh S. Altai D. (2016). Role of humic acid and amino acids in limiting loss of nitrogen fertilizer and increasing productivity of some wheat cultivars grown under newly reclaimed sandy soil. Int. J. Advanced Res. Biol. Sci. 3 (4), 123–136. doi: 1.15/ijarbs-2016-3-4-18

[B13] Khaled H. Fawy H. A. (2011). Effect of different levels of humic acids on the nutrient content, plant growth, and soil properties under conditions of salinity. Soil Water Res. 6 (1), 21–29. doi: 10.17221/4/2010-SWR

[B14] Kim B. Kim T.-H. Lee B. (2021). Optimal synthesis of carboxymethylcellulose-based composite superabsorbents. Korean J. Chem. Eng. 38 (1), 215–225. doi: 10.1007/s11814-020-0681-4

[B15] Kumar P. Sharma P. K. (2020). Soil salinity and food security in India. Front. Sustain. Food Syst. 4, 533781. doi: 10.3389/fsufs.2020.533781

[B16] Li S. Liu Z. Li J. Liu Z. Gu X. Shi L. (2022). Cow manure compost promotes maize growth and ameliorates soil quality in saline-alkali soil: Role of fertilizer addition rate and application depth. Sustainability 14 (16), 10088. doi: 10.3390/su141610088

[B17] Lima L. M. Bastos M. S. Ávila C. L. Ferreira D. D. Casagrande D. R. Bernardes T. F. (2022). Factors determining yield and nutritive value of maize for silage under tropical conditions. Grass Forage Sci. 77 (3), 201–215. doi: 10.1111/gfs.12575

[B18] Lima D. L. Santos S. M. Scherer H. W. Schneider R. J. Duarte A. C. Santos E. B.. (2009). Effects of organic and inorganic amendments on soil organic matter properties. Geoderma 150 (1-2), 38–45. doi: 10.1016/j.geoderma.2009.01.009

[B19] Masunga R. H. Uzokwe V. N. Mlay P. D. Odeh I. Singh A. Buchan D.. (2016). Nitrogen mineralization dynamics of different valuable organic amendments commonly used in agriculture. Appl. Soil Ecol. 101, 185–193. doi: 10.1016/j.apsoil.2016.01.006

[B20] Munns R. Gilliham M. (2015). Salinity tolerance of crops - what is the cost? New Phytol. 208 (3), 668–673. doi: 10.1111/nph.13519 26108441

[B21] Mustafa G. Akhtar M. S. Abdullah R. (2019). Salt stress, microbes, and plant interactions: Causes and solution (Singapore: Springer), 1–19.

[B22] Nie H. Liu M. Zhan F. Guo M. (2004). Factors on the preparation of carboxymethylcellulose hydrogel and its degradation behavior in soil. Carbohydr. Polymers 58 (2), 185–189. doi: 10.1016/j.carbpol.2004.06.035

[B23] Olad A. Zebhi H. Salari D. Mirmohseni A. Tabar A. R. (2018). Slow-release NPK fertilizer encapsulated by carboxymethyl cellulose-based nanocomposite with the function of water retention in soil. Materials Sci. Engineering: C 90, 333–340. doi: 10.1016/j.msec.2018.04.083 29853099

[B24] Page-Dumroese D. S. Ott M. R. Strawn D. G. Tirocke J. M. (2018). Using organic amendments to restore soil physical and chemical properties of a mine site in northeastern Oregon, USA. Appl. Eng. Agric. 34 (1), 43–55. doi: 10.13031/aea.12399

[B25] Pan X. Shi M. Chen X. Kuang S. Ullah H. Lu H.. (2022). An investigation into biochar, acid-modified biochar, and wood vinegar on the remediation of saline-alkali soil and the growth of strawberries. Front. Environ. Sci. 10. doi: 10.3389/fenvs.2022.1057384

[B26] Pansu M. Gautheyrou J. (2006). Handbook of soil analysis: mineralogical, organic and inorganic methods. (Berlin Heidelberg: Springer). doi: 10.1007/978-3-540-31211-6

[B27] Peth S. Horn R. Beckmann F. Donath T. Fischer J. Smucker A. (2008). Three-dimensional quantification of intra-aggregate pore-space features using synchrotron-radiation-based microtomography. Soil Sci. Soc. America J. 72 (4), 897–907. doi: 10.2136/sssaj2007.0130

[B28] Pettit R. E. (2004). Organic matter, humus, humate, humic acid, fulvic acid and humin: their importance in soil fertility and plant health. CTI Res. 10, 1–7.

[B29] Pitman M. G. Läuchli A. (2002). Salinity: environment-plants-molecules (Singapore: Springer), 3–20.

[B30] Rahman M. S. Hasan M. S. Nitai A. S. Nam S. Karmakar A. K. Ahsan M. S.. (2021). Recent developments of carboxymethyl cellulose. Polymers 13 (8), 1345. doi: 10.3390/polym13081345 33924089 PMC8074295

[B31] Robbins C. Wiegand C. (1990). “Field and laboratory measurements,” in Agricultural Salinity Assessment and Management. (New York: American Society of Civil Engineers).

[B32] Saudy H. S. Hamed M. F. Abd El-Momen W. R. Hussein H. (2020). Nitrogen use rationalization and boosting wheat productivity by applying packages of humic, amino acids, and microorganisms. Commun. Soil Sci. Plant Anal. 51 (8), 1036–1047. doi: 10.1080/00103624.2020.1744631

[B33] Siedt M. Schäffer A. Smith K. E. Nabel M. Roß-Nickoll M. Van Dongen J. (2021a). Comparing straw, compost, and biochar regarding their suitability as agricultural soil amendments to affect soil structure, nutrient leaching, microbial communities, and the fate of pesticides. Sci. Total Environ. 751, 141607. doi: 10.1016/j.scitotenv.2020.141607 32871314

[B34] Smebye A. Alling V. Vogt R. D. Gadmar T. C. Mulder J. Cornelissen G.. (2016). Biochar amendment to soil changes dissolved organic matter content and composition. Chemosphere 142, 100–105. doi: 10.1016/j.chemosphere.2015.04.087 25980657

[B35] Sun Y.-P. Yang J.-S. Yao R.-J. Chen X.-B. Wang X.-P. (2020). Biochar and fulvic acid amendments mitigate negative effects of coastal saline soil and improve crop yields in a three year field trial. Sci. Rep. 10 (1), 1–12. doi: 10.1038/s41598-020-65730-6 32488113 PMC7265530

[B36] Wang L. Sun X. Li S. Zhang T. Zhang W. Zhai P. (2014). Application of organic amendments to a coastal saline soil in North China: Effects on soil physical and chemical properties and tree growth. PloS One 9 (2), e89185. doi: 10.1371/journal.pone.0089185 24558486 PMC3928440

[B37] Wang X. Wang J. Wang J. (2021). Seasonality of soil respiration under gypsum and straw amendments in an arid saline-alkali soil. J. Environ. Manage. 277, 111494. doi: 10.1016/j.jenvman.2020.111494 33069145

[B38] Xiong S. Xiong Z. Wang P. (1996). Soil salinity in the irrigated area of the Yellow River in Ningxia, China. Arid Land Res. Manage. 10 (1), 95–101. doi: 10.1080/15324989609381423

[B39] Yang F. Antonietti M. (2020). The sleeping giant: A polymer View on humic matter in synthesis and applications. Prog. Polymer Sci. 100, 101182. doi: 10.1016/j.progpolymsci.2019.101182

[B40] Zhang J. Wang Q. Shan Y. Guo Y. Mu W. Wei K.. (2022). Effect of sodium carboxymethyl cellulose on water and salt transport characteristics of saline–alkali soil in Xinjiang, China. Polymers 14 (14), 2884. doi: 10.3390/polym14142884 35890661 PMC9316802

[B41] Zhang T. Zhan X. He J. Feng H. (2019). Moving salts in an impermeable saline-sodic soil with drip irrigation to permit wolfberry production. Agric. Water Manage. 213, 636–645. doi: 10.1016/j.agwat.2018.11.011

[B42] Zhao K. Yang Y. Peng H. Zhang L. Zhou Y. Zhang J.. (2022). Silicon fertilizers, humic acid and their impact on physicochemical properties, availability and distribution of heavy metals in soil and soil aggregates. Sci. Total Environ. 822, 153483. doi: 10.1016/j.scitotenv.2022.153483 35093361

